# Catheter-Associated Urinary Tract Infection in Critically Ill Patients: A Comparative Evaluation Between Latex Urinary Catheter and Metal Alloy-Coated Urinary Catheter

**DOI:** 10.7759/cureus.79689

**Published:** 2025-02-26

**Authors:** Farah Adibah Mohamed Hassan, Aliza Mohamad Yusof, Saw Kian Cheah, Mohd Khazrul Nizar Abd Kader, Wan Rahiza Wan Mat, Mohammad Nizam Mokhtar

**Affiliations:** 1 Department of Anaesthesiology and Intensive Care, Faculty of Medicine, Universiti Kebangsaan Malaysia, Kuala Lumpur, MYS

**Keywords:** hospital acquired infection, infection, intensive care unit, latex, metal alloy, urinary catheter

## Abstract

Background and objective

Catheter-associated urinary tract infection (CAUTI) accounts for 9% of all hospital-acquired infections (HAI). This study aimed to assess the incidence of CAUTI among patients with two different types of urinary catheters: the latex urinary catheter and the latex-coated metal alloy urinary catheter, used in the ICU setting.

Methods

This was a randomized, prospective, single-blinded study involving 76 ICU patients requiring catheterization and admitted to the ICU for more than 48 hours. Patients were allocated to the metal alloy catheter group and latex catheter group, with a randomization ratio of 1:1. CAUTI incidence was assessed at study entry, after 48 hours in the ICU, day seven in ICU, as well as any signs or symptoms of CAUTI detected during ICU stay. For statistical analysis, categorical data were compared using the chi-square or Fischer's exact test, and clinical outcomes were compared using the t-test.

Results:

A total of 76 patients were initially recruited for the study; however, six of them dropped out, leaving 70 patients for the final assessment. The mean age of the cohort was 48.6 ± 19.2 years and a majority of them were of Malay race (70.0%) and male (61.4%). The mean Acute Physiology and Chronic Health Evaluation II (APACHE II) and Sequential Organ Failure Assessment (SOFA) scores were 13.2 ± 4.0 and 6.1 ± 2.0 respectively. The mean ICU length of stay was 6 ± 1.58 days and the mean day of catheterization was 6 ± 1.54 days. One incidence of CAUTI was seen in the latex catheter group while no incidence was observed in the metal alloy group.

Conclusions

Based on our findings, there is no statistically significant difference in the incidence of CAUTI between the latex urinary catheter and the metal alloy urinary catheter groups for short-term catheterization in critically ill patients.

## Introduction

Urinary tract infection (UTI) accounts for 20% of all hospital-acquired infections (HAI) [1]. In the ICU setting, indwelling urinary catheters are frequently used to accurately measure urine output and this poses a risk of contracting a catheter-associated urinary tract infection (CAUTI), where the infection rate is approximately 9% of all HAI [2]. According to the National Healthcare Safety Network (NHSN), CAUTI is considered symptomatic when the patient meets all three following criteria: the presence of indwelling urinary catheter for more than 48 hours; one of the clinical signs (e.g., fever of more than 38 °C, suprapubic tenderness, costovertebral angle pain); and urine culture with no more than two species of organisms, at least one of which is a bacterium of ≥10^5^ colony forming units per milliliter (CFU/ml).

Positive culture does not include mixed flora, as it represents more than two organisms. Other organisms that are excluded from the CAUTI definition comprise any candida species in the urine including yeast, mold, dimorphic fungi, and parasites. On the other hand, symptomatic bacteremic urinary tract infection (SBUTI) is diagnosed in a patient who fulfills the criteria for CAUTI along with a positive blood culture with at least one matching bacteria with that of a urine culture. A diagnosis of asymptomatic bacteremic urinary tract infection (ABUTI) requires the following criteria to be fulfilled: indwelling urinary catheter in place for more than 48 hours, no signs or symptoms of CAUTI, and having organism identified from blood specimen with at least one matching bacterium to that in urine culture [3]. 

In an indwelling urinary catheter, the formation of biofilm consists of adherent microorganisms, their extracellular products, and host components deposits on the catheter. The detachment of the biofilm causes seeding, and CAUTI occurs when there is an immune response toward the biofilm. This biofilm may lead to several issues, which include difficulty of removal by shear force, resistance to phagocytosis, and resistance to antimicrobial agents [4]. Latex catheters have proven to be soft, flexible, inexpensive, and thermo-sensitive towards body temperature. However, they are associated with a risk of developing CAUTI as bacteria are more susceptible to adhering to latex [5].

Measures to combat CAUTI also include catheters with a surface that can reduce its incidence. Coated urinary catheters have been developed over the past few years and include antibiotic-coated, and those with nitrofurazone and silver or metal alloy coating [5]. The metal alloy coating is applied to the inner and outer surfaces of the catheter shaft, balloon, and tip of the device. Metal alloy works as an antiseptic by preventing the attachment of biofilm to the catheter lining. It is postulated to have a galvanic effect, which prevents microbial adhesion to its surface [6]. It is a non-releasing coating of gold, silver, and palladium and no serious adverse events have been reported related to its coating [7].

A study conducted in the ICU setting of King Fahad Hospital in Saudi Arabia has shown that metal alloy-coated urinary catheter reduces the incidence rate of CAUTI [8]. It was a single-blinded, randomized, prospective investigation that included patients using urinary catheters for three days [8]. Having a CAUTI increases the mortality, morbidity, and length of ICU stay as the risk of bacteriuria increases linearly with additional catheter days [9]. Moreover, CAUTI can lead to cystitis and pyelonephritis, which can cause bloodstream bacterial infection [10]. It also escalates the overall cost and duration of antibiotics, which can lead to antibiotic resistance [10]. Prevention and managing these risk factors can ultimately reduce the incidence of CAUTI. In light of this, we evaluated the incidence of CAUTI for short-term catheterization of less than two weeks by comparing latex and metal alloy urinary catheters in critically ill patients, in whom the risk factor of developing HAI is higher than non-critically ill patients [11]. Our secondary objective was to investigate any potential correlation between CAUTI and acute kidney injury (AKI).

## Materials and methods

This prospective, single-blinded, randomized controlled trial was conducted in the ICU, Hospital Canselor Tuanku Muhriz, Universiti Kebangsaan Malaysia (UKM) between July 1, 2023, and June 30, 2024. The study was approved by the Research and Ethics Committee, Department of Anaesthesiology & Intensive Care, and the Medical Research & Ethics Committee, UKM. The study was registered at ClinicalTrials.gov with the ID number NCT06804798. Written and informed consent was obtained from the patient or the next of kin if the patient was unable to give consent. Data collection was conducted by a single investigator. 

The defined inclusion criteria were as follows: adult patients with an indwelling urinary catheter for more than 48 hours in the ICU. The exclusion criteria were as follows: known case of urosepsis or community UTI, chronic kidney disease and end-stage renal failure, suprapubic catheter, patients with dependent urinary catheter, pregnant women, congenital urinary tract abnormality, a surgical procedure involving the urinary tract, moribund patients who were expected to die within 24 hours, or those with latex allergy.

Sample size calculation

The alpha value was set at 0.05 and a power of 80% was adopted. The sample size was calculated using Epi Info 7 according to Fleiss (1981) formula [12]. Based on a previous study by Aljohi et al., there was a significant difference in the rate of CAUTI between the standard catheter and noble metal alloy catheter (33 % vs. 3.3% per catheter days) [8]. Therefore, the total sample size needed was 76 patients, factoring in a potential 20% dropout rate.

Study design

Patients recruited for the study were randomized into two groups. Group A (the control group) received the latex catheter and Group B (the intervention group) received the latex-covered metal alloy catheter. The process of randomization was done using computer-generated code, with 1:1 randomization. Depending on the randomization, Group A or Group B type of catheter was handed over by the primary investigator to the healthcare personnel for the insertion.

**Figure 1:**
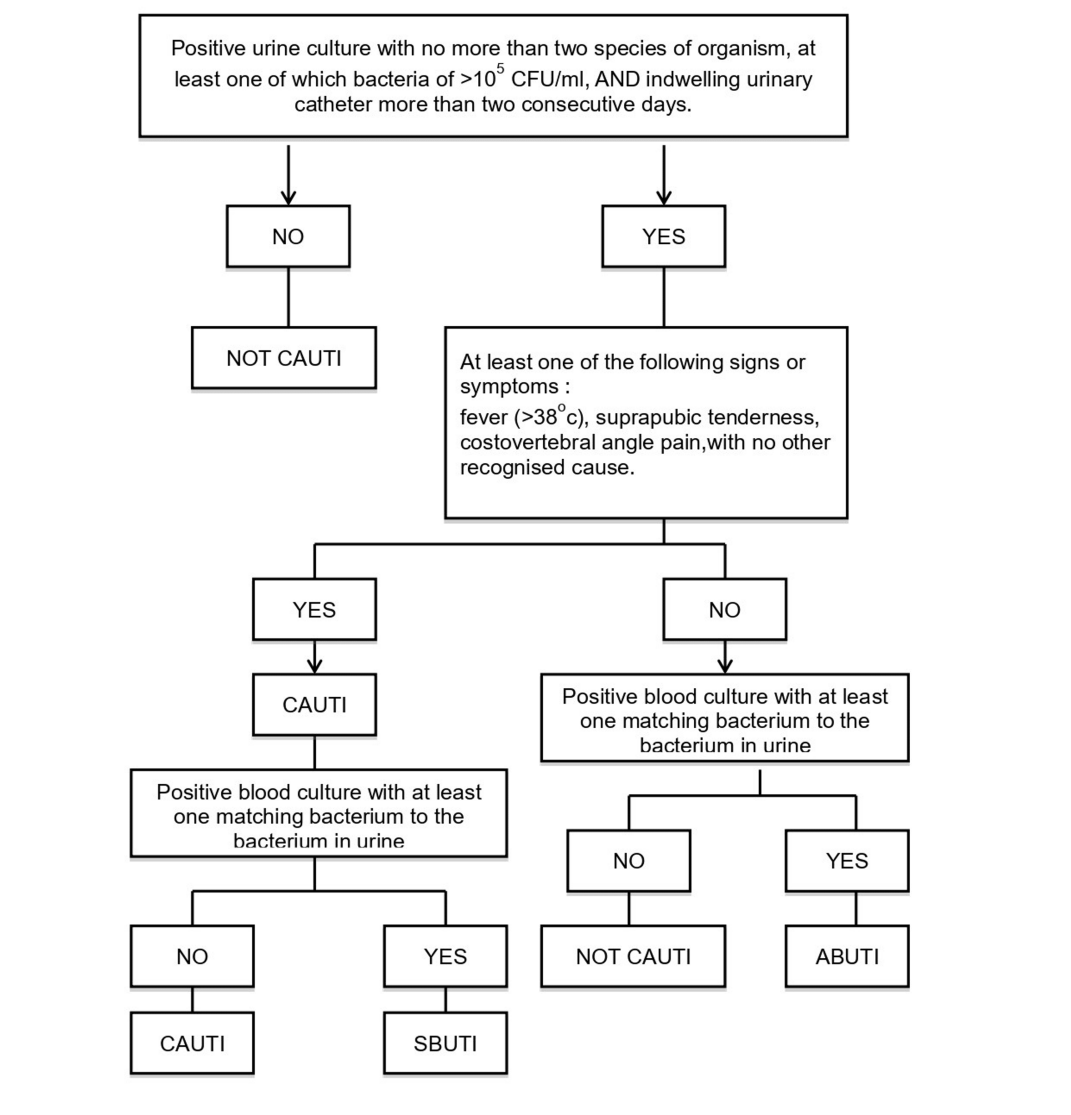
Criteria for diagnosing CAUTI ABUTI: asymptomatic bacteremic urinary tract infection; CAUTI: catheter-associated urinary tract infection; SBUTI: symptomatic bacteremic urinary tract infection

**Figure 2:**
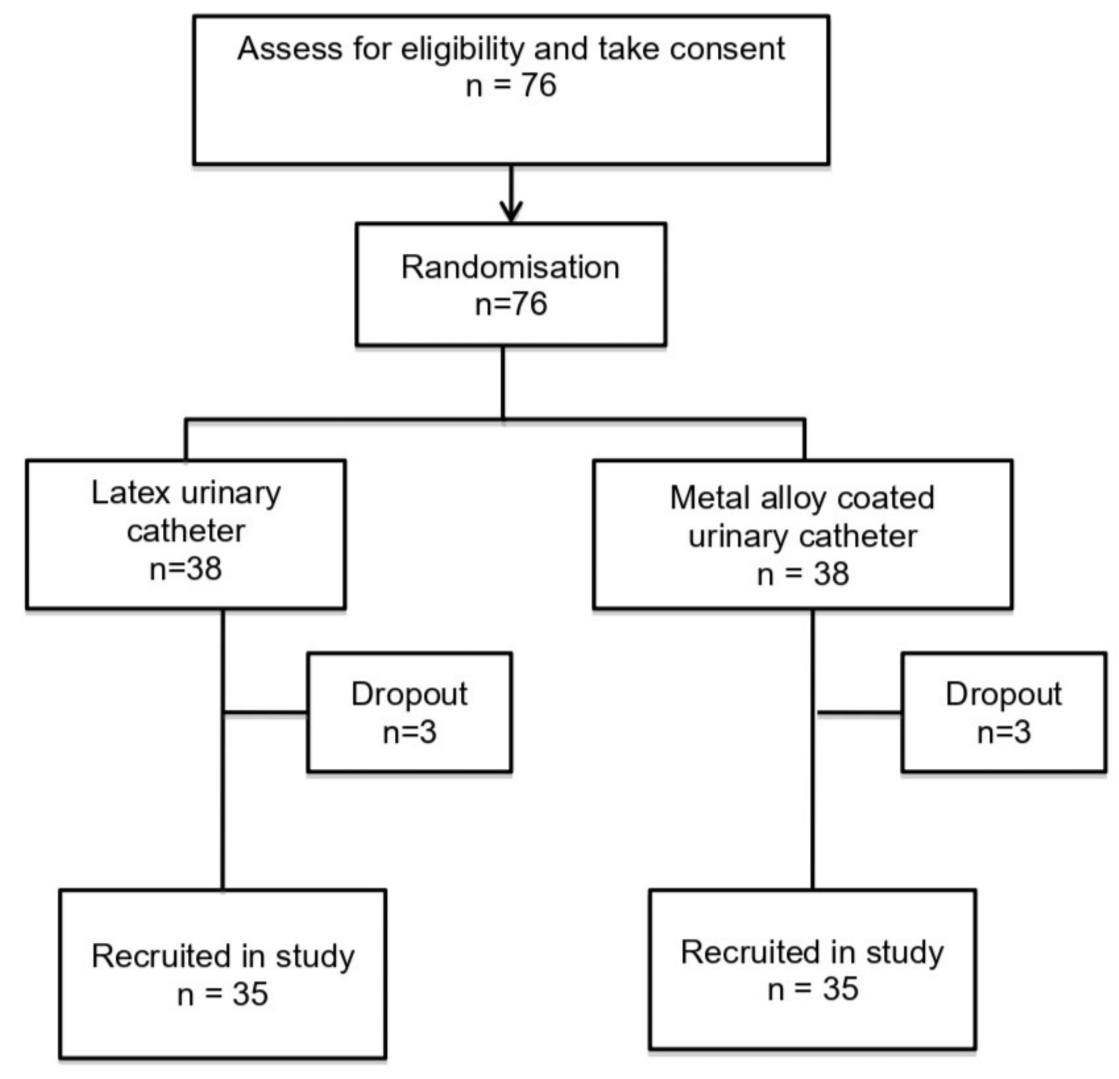
Flow chart depicting patient recruitment

A signed consent from either patient or relative was obtained before any recruitment of patients. Patients who were admitted to the ICU without a urinary catheter would be given the urinary catheter based on randomization and the day of the catheter insertion would be considered day one. The same applied to patients who already had a urinary catheter in situ; the preexisting urinary catheter was taken off and a randomized catheter was given. Before the insertion of the randomized catheter, a urine sample was taken to exclude community UTI, symptomatic urinary tract infection (SUTI), or CAUTI from a previous catheter. If the result of the urine culture was negative, the patient was enrolled and the day of catheter insertion was deemed as day one. However, the patient was considered a dropout if the culture was positive. Figure 1 illustrates the criteria to define CAUTI and Figure 2 presents a flowchart depicting the selection process.

The urinary catheter was inserted under an aseptic technique with a closed drainage system. Demographic data included age, gender, patient's comorbidities, diagnosis for which the presented before urinary catheter placement, as well as Acute Physiology and Chronic Health Evaluation II (APACHE II) and Sequential Organ Failure Assessment (SOFA) scores, and these parameters were recorded. Clinical data included total duration of the catheter, antimicrobial coverage, urine output over 24 hours, signs and symptoms of CAUTI such as fever of more than 38 °C, suprapubic tenderness, and costovertebral angle pain were elicited in conscious patients. Laboratory data included urinary culture, blood culture, and daily creatinine levels. The staging of AKI was recorded according to the Kidney Disease: Improving Global Outcomes (KDIGO) classification. The requirement for dialysis from AKI was also documented. As for blood and urine culture, it was collected on days 3, 7, and 14 of catheter insertion to rule out ABUTI or when the patient developed CAUTI during their ICU stay. All data mentioned above were collected from the day of urinary catheterization in the ICU until that patient was discharged or declared dead or a maximum of 14 days in ICU or until the patient developed CAUTI or ABUTI, whichever occurred earlier. Patients who died within 48 hours of catheter insertion, or were discharged or requiring dialysis within that time, were also considered dropouts. All data were collected by adhering to the ethical principles stipulated in the Declaration of Helsinki 2008.

Statistical analysis

Data were analyzed using SPSS Statistics version 28.0 (IBM Corp., Armonk, NY). The distribution of continuous variables was explored using skewness, kurtosis, and histograms. Continuous variables were reported as mean ± standard deviation (SD) if normally distributed; otherwise, they were presented as median (25th percentile, 75th percentile). Categorical variables were presented as frequencies and percentages. The differences in outcomes between patients who received latex catheters or metal alloy-coated catheters were explored using an Independent sample t-test, Mann-Whitney U test, Pearson chi-squared test, and Fisher's exact test. All the tests were two-sided and statistical significance was denoted by p<0.05.

## Results

A total of 76 patients were initially recruited for the study; however, six dropped out, leaving 70 patients for the final analysis. Of these, 35 patients were assigned to the latex group, and 35 patients to the metal alloy group. Of the six dropouts, four required dialysis within 48 hours of admission, one patient had a positive urinary culture upon ICU admission, and one patient was discharged from ICU within 48 hours of admission. The demographic parameters of the two groups are demonstrated in Table 1. The reported mean age was 48.60 ± 19.28 years old, with a majority of them belonging to the Malay race (70.0%) and male (61.4%). The median BMI was 24.5 kg/m^2^. Reported comorbidities included diabetes mellitus (20%), hypertension (42.9%), ischemic heart disease (IHD) (5.7%), malignancy (5.7%), chronic obstructive pulmonary disease (COPD) (1.4%), bronchial asthma (2.9%), smoking (11.4%), and obesity (5.7%). The mean APACHE II and SOFA scores were 13.27 ± 4.08 and 6.14 ± 2.02 respectively.

**Table 1:** Comparison of demographic parameters between the two groups ^a^Independent sample t-test. ^b^Mann-Whitney U test. ^c^Pearson chi-squared test. ^d^Fisher's exact test APACHE: Acute Physiology and Chronic Health Evaluation; BMI: body mass index; IQR: interquartile range; SD: standard deviation; SOFA: Sequential Organ Failure Assessment

Variables		All (n=70)	Latex (n=35)			Metal alloy (n=35)	P-value
Age, years, mean ± SD		48.60 ± 19.28	50.03 ± 21.19			47.17 ± 17.36	0.539^a^
Gender, n (%)							0.027^c^
Female		27 (38.6)	18 (51.4)			9 (25.7)	
Male		43 (61.4)	17 (48.6)			26 (74.3)	
Race, n (%)							0.083^d^
Malay		49 (70.0)	23 (65.7)			26 (74.3)	
Chinese		18 (25.7)	12 (34.3)			6 (17.1)	
Indian		3 (4.3)	0 (0.0)			3 (8.6)	
BMI, kg/m^2^, median (IQR)		24.51 (22.38,27.13)	23.88 (21.48, 25.71)			25.39 [23.53, 27.34]	0.118^b^
Diabetes mellitus, n (%)							0.550^c^
No		56 (80.0)	29 (82.9)			27 (77.1)	
Yes		14 (20.0)	6 (17.1)			8 (22.9)	
Hypertension, n (%)							0.334^c^
No		40 (57.1)	18 (51.4)			22 (62.9)	
Yes		30 (42.9)	17 (48.6)			13 (37.1)	
Smoker, n (%)							>0.950^d^
No		62 (88.6)	31 (88.6)			31 (88.6)	
Yes		8 (11.4)	4 (11.4)			4 (11.4)	
Ischemic heart disease, n (%)							>0.950^d^
No		66 (94.3)	33 (94.3)			33 (94.3)	
Yes		4 (5.7)	2 (5.7)			2 (5.7)	
Obesity, n (%)							0.614^d^
No		66 (94.3)	34 (97.1)			32 (91.4)	
Yes		4 (5.7)	1 (2.9)			3 (8.6)	
Malignancy, n (%)							>0.950^d^
No		66 (94.3)	33 (94.3)			33 (94.3)	
Yes		4 (5.7)	2 (5.7)			2 (5.7)	
Chronic obstructive pulmonary disease, n (%)							>0.950^d^
No	69 (98.6)			35 (100.0)	34 (97.1)		
Yes	1 (1.4)			0 (0.0)	1 (2.9)		
Bronchial asthma, n (%)							>0.950^d^
No	68 (97.1)			34 (97.1)	34 (97.1)		
Yes	2 (2.9)			1 (2.9)	1 (2.9)		
APACHE II score, mean ± SD		13.27 ± 4.08	14.20 ± 4.22			12.34 ± 3.76	0.056^a^
SOFA score, mean ± SD		6.14 ± 2.02	6.71 ± 1.93			5.57 ± 1.96	0.017^a^

The metal alloy group had a higher proportion of males compared to latex (17 (48.6%) vs. 26 (74.3%); p=0.027). Besides, the metal alloy group was observed to have a significantly lower mean SOFA score compared to the latex group (6.71 ± 1.93 vs. 5.57 ± 1.96; p=0.017). No significant differences were observed in other demographic characteristics between the two groups (p>0.05).

**Figure 3:**
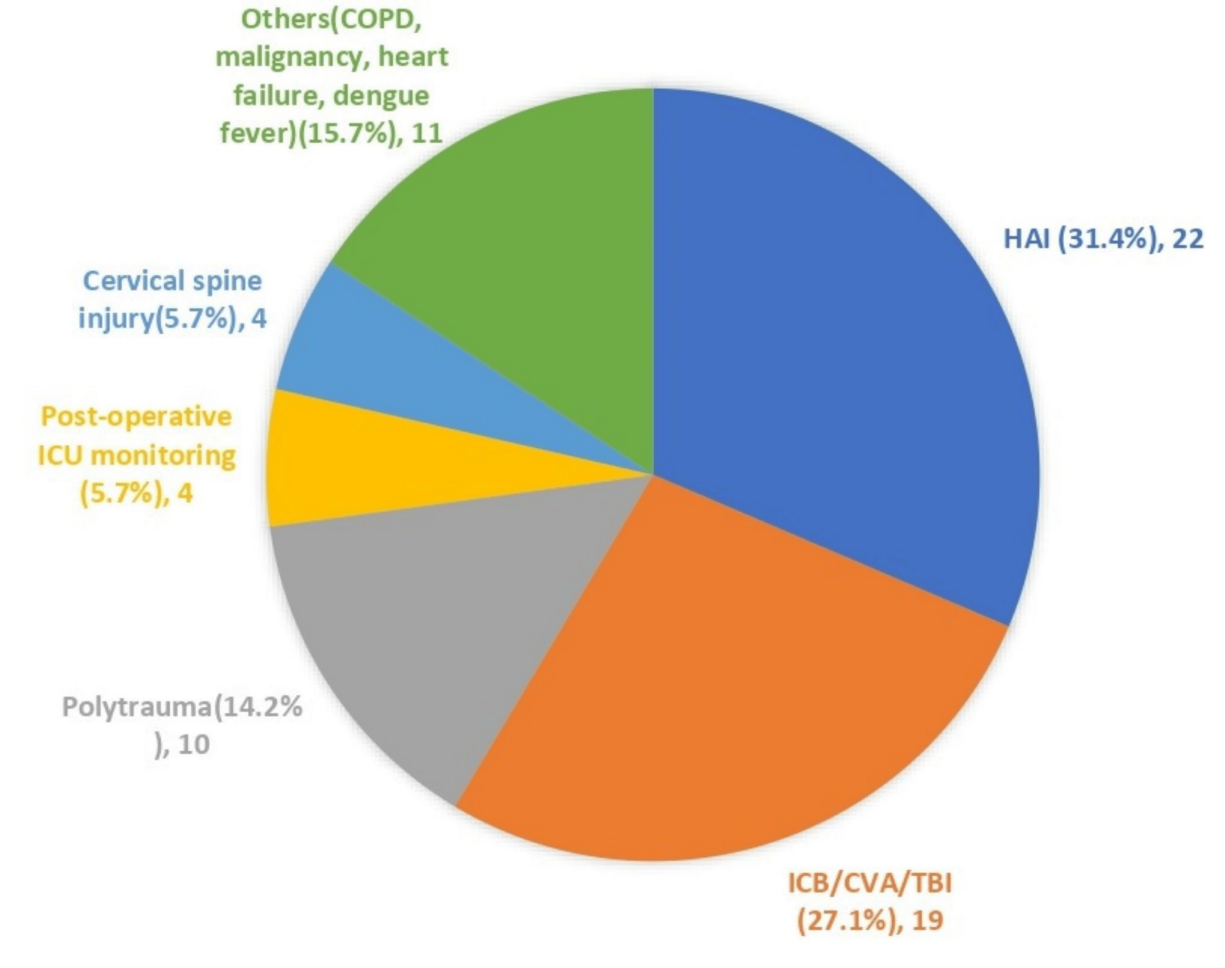
Pie chart showing diagnosis on admission COPD: chronic obstructive pulmonary disease; CVA: cerebrovascular accident; HAI: hospital-acquired infection; ICB: intracranial bleed; ICU: intensive care unit; TBI: traumatic brain injury

HAI was the main diagnosis on admission among the 70 patients recruited, accounting for 34.1%, followed by brain injury (21.7%) and polytrauma (14.2%), as shown in Figure 3.

The clinical outcomes between the two groups are compared in Table 2. One patient died in the ICU, and the mean ICU length of stay was 6 ± 1.58 days. Five AKI cases were reported on day three: one (1.4%) with AKI stage 2 (in the metal alloy group) and four (5.7%) with AKI stage 3 (three in the latex group, and one in the metal alloy group). One CAUTI case was reported, on day three, from the latex group. The mean duration of catheterization was 6 ± 1.54 days. No significant differences were observed in clinical outcomes between the two groups (p>0.05).

**Table 2:** Comparison of clinical outcomes between the groups ^a^Independent sample t-test. ^b^Fisher's exact test AKI: acute kidney injury; CAUTI: catheter-associated kidney injury; ICU: intensive care unit; SD: standard deviation

Variables	All (n=70)	Latex (n=35)	Metal alloy (n=35)	P-value
ICU outcome, n (%)				>0.950^b^
Alive	69 (98.6)	34 (97.1)	35 (100.0)	
Dead	1 (1.4)	1 (2.9)	0 (0.0)	
Length of ICU stay, days, mean ± SD	5.97 ± 1.58	6.20 ± 1.57	5.74 ± 1.58	0.228^a^
Day 3 AKI staging, n (%)				
Stage 2	1 (1.4)	0 (0.0)	1 (2.9)	
Stage 3	4 (5.7)	3 (8.6)	1 (2.9)	
Day 3 CAUTI, n (%)	1 (1.4)	1 (2.9)	0 (0.0)	
Day 7 AKI staging, n (%)	0 (0.0)	0 (0.0)	0 (0.0)	
Day 7 CAUTI, n (%)	0 (0.0)	0 (0.0)	0 (0.0)	
Days of catheterization, mean ± SD	5.87 ± 1.54	6.00 ± 1.52	5.74 ± 1.58	0.489^a^

## Discussion

CAUTI is one of the most common HAI and it is the second most common HAI in the United States of America and Southeast Asia respectively [13]. Of note, 80% of UTIs are attributable to indwelling urinary catheter use, and up to 79% of indwelling urinary catheters are used in the ICU setting [14].

In this study, 70 ICU patients were recruited: 22 patients (31.4%) were found to have HAI, which aligns with the prevalence rate in another study (22%) [14]. There was only one documented case of CAUTI (in the latex catheter group). Risk factors for developing CAUTI include the length of catheterization (more than 14 days), inappropriate antibiotic use, immunosuppressive drugs, female gender, advanced age (60-80 years old), stroke, and paraplegia [15,16]. The lower-than-expected CAUTI incidence observed in our study can be attributed to the shorter mean catheterization duration (six days), which may not have been sufficient for significant bacterial colonization and biofilm formation; there was almost no difference in the incidence of CAUTI between the latex and metal alloy groups in the short term, similar to a study by Pickard et al. where, instead of metal alloy catheter, they compared silver-coated metal alloy catheter with nitrofural impregnated catheter [17]. Another study by Tasseau et al. showed that the risk of CAUTI increased from 19% to 50% from day five to day 14 of catheterization [18].

The reported risk factors for CAUTI were mostly present in our study. A 70-year-old male patient, with no known medical illness who had a cervical fracture and tetraplegia, developed CAUTI on day three of catheterization. Although the female gender has a stronger predisposition for developing CAUTI, one study has reported males having CAUTI more than females, with a ratio of 1:1.12 [19]. In our study, this may be due to more males being recruited and catheterized than females (61%). However, in the study by Saleem et al., males were more predisposed to CAUTI, perhaps due to the physiological prostate changes in elderly men, which make them vulnerable to UTI. Furthermore, the use of a size 16Fr (French) catheter may cause damage to the urethra and bladder neck, causing bladder spasms, which predispose individuals to UTI [19].

Gram-negative bacteria were the main causative agent for CAUTI (63%), followed by Gram-positive bacteria, which shows Enterococcus sp. as the primary causative microorganism (62%) [13]. In our study, Enterococcus faecalis was isolated in the positive CAUTI result on day three. The patient was already on intravenous ceftriaxone two days before the catheterization. Once CAUTI was confirmed, the latex catheter was removed and changed, and intravenous vancomycin was started as targeted therapy. CAUTI, which is associated with gram-negative bacteria like Enterococcus faecalis, is commonly acquired via the extraluminal route where inoculation can occur early by direct contamination when the catheter is inserted, or later by the organisms ascending the perineum [13].

Furthermore, multiple studies also show that 10-15% of CAUTI is caused by Candida species (Yeast sp.) and candiduria is more common in ICU [20]. In our study, there were four reported cases of yeast in urine culture: three in the latex group and one in the metal alloy group, accounting for 5% of the recruited patients. Before 2015, the NHSN included yeast as part of the CAUTI definition. Subsequent studies indicate that while yeast is a rare cause of UTI, catheter colonization is relatively common. Furthermore, the treatment of candiduria has not been shown to provide clinical benefit, and including yeast in diagnostic criteria can lead to inappropriate antifungal prescribing [21]. However, this issue is largely mitigated by the 2024 NHSN CAUTI definition, which excludes Candida, yeast, dimorphic fungi, molds, and parasites [3]. The ICU condition, host factors, patient population, prior antimicrobial exposure, and the organisms are unique to each ICU facility and may explain the differences in the findings of microorganisms of CAUTI [22]. A high proportion of patients in our study received antibiotic therapy before or during catheterization, which may have suppressed bacterial colonization in the urinary tract, further reducing the observed incidence of CAUTI.

To reduce the rate of CAUTI, the prevention of modifiable risk factors and preventative measures are key. Modifiable risk factors include indication of urinary catheter insertion, timing of discontinuation, and appropriate antibiotic usage. A physician’s order is required and the institution should document the time of catheter placement [23]. In our ICU setting, once the order is received, the nursing staff will document the time and size of the catheter in a separate folder for each patient. This is important as it has helped reduce the risk of CAUTI in our ICU. Other measures include the insertion of the catheter by trained healthcare workers by adhering to the aseptic technique. Furthermore, it has been shown that the closed urinary catheter collection system reduces the risk of CAUTI [23].

We were unable to investigate the association between CAUTI and AKI due to a lack of available data. However, only a few studies have shown that UTI may lead to AKI concerning the pathogenesis of sepsis and septic shock. Patients who have UTI and are more at risk of developing AKI include those of older age, those with diabetes mellitus, a higher baseline estimated glomerular filtration rate (eGFR), fever, and those on nephrotoxic medications [24].

Measures to avoid the incidence of CAUTI and to define it are still being studied and developed [25,26]. In the ICU setting, where most of the critically ill patients are intubated, symptoms of CAUTI are very rarely elicited, making the diagnosis of CAUTI difficult. Almost half of patients (51%) have other contributing factors for fever, such as pneumonia [25]. A more efficient diagnostic marker and faster way that is being researched to define CAUTIs pertains to biomarkers. Potential biomarkers that are studied include interleukins (IL-6, IL-8), lactoferrin, and immunoglobulin A (IgA) [26,27]. General ways to reduce the incidence of CAUTI include the implementation of a CAUTI bundle in hospitals, which includes catheter insertion, management, and surveillance, as well as educating staff regarding this. Other ways that are being studied include catheter linings and coatings.

Nanoparticles, particularly gold, are being investigated, concerning their mechanism of action of disruption of membrane potential and reduction of adenosine triphosphate (ATP) in bacteria. More studies are being conducted regarding its safety and efficacy. Bacteriophages-impregnated catheters are extremely specific and hence less likely to damage healthy gut or vaginal flora [26,28]. However, bacteria can evolve its receptors and become resistant to these bacteriophages. Furthermore, bacterial interference is promising, and clinical trials are ongoing. This method uses the native flora to outcompete pathogenic bacteria for colonization [26]. Engineered bacteria is used to compete with colonizer bacteria, thereby blocking the formation of biofilm. Vaccination strategies for preventing UTI are also underway and the principles of this vaccine can also be used for CAUTI as studies have shown that they target similar bacteria. However, it is still debatable whether these can be a stand-alone or complementary strategy to combat CAUTI [26].

This study has several limitations. Firstly, it involved a relatively small sample size given a limited time frame where the mean turnover rate in this ICU was less than 10 days. Furthermore, there is no data available regarding CAUTI in our ICU and no pilot study has been done on the topic. Secondly, this was a single-center study and hence the findings might not be generalized to other hospitals. Also, the diagnosis of CAUTI itself is a thorny issue as specific diagnostic criteria are still a matter of debate worldwide. The criteria and diagnostic tools vary in different CAUTI definitions and guidelines [29].

## Conclusions

This study found no statistically significant difference in the incidence of CAUTI between the latex urinary catheter and the metal alloy-coated urinary catheter in critically ill patients requiring short-term catheterization. Although only one CAUTI case was recorded (in the latex catheter group), the overall low incidence limits the ability to draw definitive conclusions on the superiority of one catheter type over the other. Given the potential benefits of metal alloy coatings in reducing biofilm formation and bacterial adhesion, further studies with larger sample sizes and longer observation periods are warranted to assess their efficacy more comprehensively. We believe this study would pave the way for future research exploring the impact of coated catheters on infection rates beyond ICU settings, to delve into their cost-effectiveness, patient outcomes, and potential role in antimicrobial stewardship.
